# Leveraging pharmacy partnerships and Grant funding to improve access to medications for opioid use disorder

**DOI:** 10.1016/j.rcsop.2025.100653

**Published:** 2025-09-13

**Authors:** John M. Vasko, Erin Shirley Orey, Carolanne Wartman, James K. Rowlett, Jefferson D. Parker, Donna M. Platt, Julie A. Schumacher

**Affiliations:** a University of Mississippi Medical Center, Jackson, MS 39216, United States; b The University of Mississippi School of Pharmacy, Jackson, MS 39216, United States; c The University of Arizona College of Pharmacy, Tucson, AZ 85721, United States; d Prevention Research Institute, Lexington, KY 40522, United States

**Keywords:** Pharmacy, Opioid use disorder, Medications for opioid use disorder, Rural health disparities, Collaboration, Treatment access

## Abstract

**Background:**

Increased availability and access to FDA-approved medications for opioid use disorder (MOUD) can improve efforts to address the ongoing opioid public health crisis. Pharmacist collaboration is essential yet often inconsistent in MOUD access. This unpredictability creates a treatment bottleneck, especially in rural areas.

**Objective:**

To investigate the reasons for independent pharmacies' reluctance to collaborate with the Mississippi Horizons Project, a program designed to improve patient access to MOUD.

**Methods:**

A Horizons team member contacted pharmacies using a script to inquire about accepting payment for a patient's MOUD prescription. Pharmacist responses about collaboration (i.e., collaborated or denied collaboration) after initial contact and follow-up were summarized. A single coder inductively derived categories within which responses about collaboration were totaled.

**Results:**

After initial contact and follow-up, only slightly more than half of pharmacies contacted (53.5 %) collaborated with the Horizons project to accept payment for and dispense MOUD. Categories for non-collaboration included regulatory misperceptions, unfamiliarity with patients and providers, and payment and logistical barriers. Successful efforts for collaboration after initial contact included utilizing existing relationships with pharmacies and providing additional information about the Horizons project.

**Conclusion:**

Integrating educational interventions with structural and policy measures, while fostering collaborative partnerships with pharmacies and other key stakeholders, can help increase familiarity, reduce stigma, address operational challenges, and enhance cooperation to increase pharmacy collaboration and MOUD access to patients in rural areas and nationwide.

## Highlights

• Several barriers exist for MOUD dispensing among Mississippi pharmacies.
• Slightly less than half of contacted pharmacies declined to collaborate.
• Misperceptions about MOUD dispensing were most commonly cited.
• Peer-to-peer and educational interventions could increase collaboration.

## 1 Introduction

The opioid epidemic is a public health crisis that has contributed to rates of substance use disorders and opioid use-related deaths. In the United States, the economic burden of the opioid epidemic (e.g., lost productivity, reduced quality of life, increased usage of the health care system) exceeds $1 trillion annually.^1^ Nationally, opioids contributed to a significant and increasing number of overdose deaths,2., 3., 4, 5. with deaths involving synthetic opioids such as fentanyl becoming more common. In Mississippi, opioid use is an alarming issue. In 2021, 70 % of overall overdose deaths involved opioids. Of these deaths, 85 % involved fentanyl.^6^ Further, opioid overdose deaths have increased in Mississippi by 630 % in the past decade.^6^ This recent elevation in opioid overdose deaths in Mississippi compared to previous state-level trends highlights a critical need for intervention.

A front-line treatment approach for opioid use disorder (OUD) is medication for OUD (MOUD).^7^ These medications can be used as standalone treatments for OUD^8^ and are intended to reduce opioid cravings, withdrawal symptoms, and overdose deaths.8., 9., 10. Compared to alternative treatment approaches (e.g., psychosocial interventions alone), MOUD is associated with a greater magnitude of and more sustained treatment gains, such as reduced opioid overdose deaths.^11^^,^^12^ FDA-approved MOUD includes methadone, naltrexone, and buprenorphine (alone or formulated with naloxone). Methadone is only available at federally certified opioid treatment programs and acute inpatient hospital settings.^9^^,^^13^ In contrast, both oral buprenorphine and naltrexone can be dispensed at a pharmacy with a prescription.^14^, 15., 16. Both state-level data for Mississippi and national data suggest potential barriers to access for MOUD.^17^^,^^18^ Further, about 30 % of MOUD prescriptions are not filled within 30 days of being written for a patient.^19^ Such data indicate broader treatment gaps involving accessing and dispensing prescribed MOUD.

Pharmacists can be gatekeepers to accessing prescribed MOUD as they are key stakeholders who dispense MOUD to patients. Pharmacies are typically easily accessible geographically and are frequently visited. Over 90 % of Americans live within two miles of a pharmacy.^20^ Moreover, patients visit pharmacies twice as often as other medical providers, such as physicians.^21^ In addition to accessibility, pharmacists' training in medication safety, management, and monitoring equips them to help patients engage in treatment. Pharmacists can be crucial in rural areas and states, such as Mississippi, that typically have fewer available options for OUD treatment.^22^

Despite the important role of pharmacists in facilitating OUD treatment, several barriers hinder their capacity to support patient access to MOUD.^23^ These barriers may be especially prevalent in Southern states, such as Mississippi, that are seemingly less willing to prescribe MOUD compared to non-Southern states.^13^ One significant barrier is regulatory misperceptions.^24^ Pharmacists may believe that there are regulatory limits and challenges in accessing and dispensing MOUD. The laws enforced by a state's board of pharmacy, such as a need for extensive record keeping and compliance with the state's prescription monitoring program, can unintentionally decrease the willingness of a pharmacist to dispense MOUD to patients even with a prescription. Pharmacists may not want to risk potential scrutiny from regulatory bodies such as the Drug Enforcement Agency (DEA) for prescribing MOUD to patients. Pharmacists may also mistakenly perceive limits to the amount of MOUD that can be ordered, stocked, and dispensed, despite no limits existing.^25^

Pharmacist attitudes toward MOUD prescribers and patients can also decrease willingness to dispense these medications. Pharmacists may be unfamiliar with patients and prescribers who are not local. Stigma toward MOUD exists, including a belief that MOUD is “replacing one substance with another,” rather than receiving actual treatment for OUD.^26^^,^^27^ These beliefs can create mistrust among pharmacists about the motives of patients who receive a prescription for MOUD. Recent policy further extends the potential mistrust of prescribers held by pharmacists. For example, an exemption to the Ryan Haight Online Pharmacy Consumer Protection Act of 2008 allows healthcare providers to prescribe MOUD via telehealth without an in-person evaluation. Although intended to provide increased access to care, the exemption may fuel or create mistrust or uncertainty among pharmacists about who is prescribing the medication, for whom it is prescribed, and the reasons for the prescription.^28^

Finally, logistical issues, such as pharmacy policies regarding acceptable payment, can interfere with MOUD access. In general, financially disadvantaged patients who are unable to self-pay or use private insurance have increased difficulties obtaining prescribed medications,^29^ which is crucial for successful OUD treatment. In Mississippi, only 5 % of prescriptions were covered by Medicare or Medicaid.^17^ Additionally, Mississippi has one of the lowest rates of MOUD for patients with Medicaid compared to other states.^30^ Furthermore, most patients in Mississippi struggle to obtain MOUD on a long-term basis. Only 7.3 % of patients sustained a 90-day continuous supply of buprenorphine during each quarter in 2018.^31^ Finally, certain pharmacies across the state do not accept payment over the phone. This can hamper efforts from federally funded initiatives that can pay for MOUD for financially disadvantaged patients.

## 2 Description of current project to expand access to MOUD in Mississippi

Initiatives that help provide access to MOUD can improve treatment outcomes for patients with OUD. The Mississippi Horizons Project, administered through the University of Mississippi Medical Center (UMMC)’s Center for Innovation and Discovery in Addictions (CIDA), was funded by a Congressional Directive Funding appropriation in the fiscal year (FY) 2022 Federal Budget administered through the Substance Abuse and Mental Health Services Administration (SAMHSA). Among the various approaches of the Horizons Project to treating substance use disorders, the initiative provides telehealth-based MOUD services to patients and covers the costs of healthcare services, including transportation and medications.

Pharmacist reluctance about dispensing MOUD can exist for various reasons (e.g., regulatory misperceptions). This reluctance can create a treatment bottleneck. Understanding reluctance is particularly useful for improving MOUD access within the context of a national opioid epidemic. As implementation of the current project began, many pharmacies were reluctant or unwilling to collaborate with the Horizons Project. Thus, the current project reviews the process of contacting pharmacies and catalogs reasons provided by pharmacists to understand unwillingness or reluctance to collaborate with the Horizons Project. The examination of these reasons for unwillingness or initial reluctance can inform future initiatives and treatment providers about pharmacist attitudes and MOUD dispensing behaviors that may be encountered. Furthermore, the current project examines the reasons provided by pharmacists who initially declined collaboration and later agreed to collaborate after additional follow-up. The examination of these reasons provides novel insight into how collaboration with community pharmacists can be improved to increase access to MOUD.

## 3 Methods

Patients were referred to the Horizons project from inpatient and outpatient settings. This included referrals from Horizons project staff who identified patients within the healthcare system in need of services, other Horizons patients who referred friends and family, medical center staff including psychologists and emergency department physicians, therapeutic family courts, other substance use treatment programs, family, friends, and self-referral based on advertisements or word of mouth. Once referred, enrollment in the program was discussed with Horizons project staff. Services provided by the Horizons project were discussed with the enrollee, including prescribing MOUD by a provider and identifying whether covering the costs of MOUD would be a need for a specific enrollee. Initially, patients were prescribed either a combination of buprenorphine and naloxone or buprenorphine. As the project progressed, only the combination of buprenorphine and naloxone was prescribed to deter diversion.

The current project describes efforts to understand the reasons for pharmacy unwillingness or initial reluctance to collaborate with the Horizons project, employing a qualitative and exploratory approach based on call notes to pharmacies. Institutional Review Board (IRB) approval was not required for the Horizons project because it was a services project, and not a research project. The project is based on notes from February 2023 through March 2023, when a Horizons team member at UMMC contacted independent pharmacies to inquire about accepting payment from the program for enrollees' medication. When a patient was enrolled in the program, a Horizons project team member obtained the patient's address and conducted a Google search of pharmacies within a 10-mile radius of the patient. The Horizons project could only pay for MOUD with a credit card over the phone. Major chain pharmacies did not accept credit card payment over the phone. This payment policy was verified by contacting several outlets in each chain. Accordingly, only reasons provided by independent pharmacies are discussed in the current study. About two-thirds of independent pharmacies across the areas included in Google searches were contacted.

A script was used when contacting independent pharmacies. Once on the phone with a pharmacist, a brief description of the Horizons project was provided, including the mechanism by which the project would pay for patients' medication. Briefly, the pharmacist was asked if they carry the MOUD, if they accept credit card payments over the phone, and if they could fax a copy of the receipt to the Horizons team member. If all conditions were agreed to, the pharmacist was informed that the prescriber would send the patient's prescription to the pharmacy. If a pharmacy denied initial collaboration, additional follow-up occurred while additional pharmacies within a 10-mile radius of the patient were contacted about collaboration. Follow-up was not scripted. Follow-up calls were completed by a Horizons team member at UMMC, who made initial contact with pharmacies, and by a therapeutic family court representative.

Pharmacist responses were summarized on an encrypted spreadsheet and maintained on secure servers accessible only to project personnel who required access for the daily functions of the project. The single coder who derived categories was provided with a deidentified spreadsheet. Pharmacist responses included an agreement to collaborate, reasons for denying collaborating (e.g., “Not taking new customers,” “They have reached their Buprenorphine limit.”), and reasons for agreeing to collaborate on follow-up. Responses for denying collaboration were categorized by one of the authors. Categories were inductively derived by a single coder and informed by existing literature on common reasons for pharmacist reluctance to dispense MOUD. Pharmacists sometimes provided more than one reason for denying collaboration. Each reason was placed within a category. Double coding, formal or secondary thematic coding, or validation did not occur due to resource constraints.

## 4 Results

Over two months, 43 pharmacies were contacted. Table 1 provides the total number of pharmacies that agreed to collaborate initially, agreed to collaborate after follow-up, and denied collaboration. Initially, 15 (34.9 %) pharmacies agreed to collaborate with the Horizons project. Although nearly two-thirds (65.1 %) of pharmacies initially declined to collaborate with the Horizons project, about one-third of those pharmacies agreed to collaborate with the Horizons project upon follow-up. Ultimately, 23 out of the 43 independently owned pharmacies contacted (53.5 %) agreed to collaborate with the Horizons project after either initial contact or follow-up.

**Table 1:** Pharmacy agreement to collaborate with the Horizons project (*n* = 43).

**Group**	**Frequency**
Collaborated from the first point of contact	15 (34.9 %)
Did not initially collaborate but joined after follow-up	8 (18.6 %)
Declined involvement despite follow-up	20 (46.5 %)

Responses provided by pharmacies for not collaborating with, as well as eventually agreeing to collaborate with, the Horizons project were categorized and tallied. The frequency of each reason is based on the categorization of each response and not based on structured interviews or coded transcripts. If a pharmacy gave multiple responses for not collaborating, each response was counted separately within its respective category. For example, one pharmacy listed the following reasons for non-collaboration: not accepting new patients, reaching the allowable dispensing limit for the medication, and only filling prescriptions from local providers. Each reason was counted toward the total count for its respective category (see Table 2). These counts are intended to help illustrate which reasons appeared more common compared to others.

**Table 2:** Reasons for denying collaboration with the Horizons project.

***Category* / Reason**	**Frequency**
*Regulatory Misperceptions*	
Reached allowable limit for buprenorphine	7
Prescriber needed to be local	6

*Unfamiliar Patients and Providers*	
Not taking new customers	4
Not accepting telehealth appointment prescriptions	2
All medications needed to be filled by the pharmacy	1

*Payment and Logistics*	
Do not accept credit card payment over the phone	3
Do not carry the specified medication	2
Out of stock of the specified medication	1

## 5 Reasons that pharmacies denied collaborating with the Horizons project

### 5.1 Regulatory misperceptions

The most cited reason for not collaborating with the Horizons project was that the pharmacy had reached the allowable dispensing limit for the specified medication for buprenorphine (e.g., “We have reached our Buprenorphine limit.”). This reason was cited by seven pharmacies.

Six pharmacies stated that the prescriber needed to be local, or the prescriber was too far from the pharmacy. Explanations for only accepting prescriptions from a local provider included concerns about the DEA (cited by two of the six pharmacies), the potential for patients misusing the MOUD (cited by one of the six pharmacies), and not knowing the prescribing patterns of non-local prescribers (cited by one of six pharmacies). One pharmacist stated, “Your provider does not live within a 40-mile radius.” Another pharmacist stated, “We only take prescriptions from local doctors.”

### 5.2 Unfamiliarity with patients and providers

Several reasons for denying collaboration with the Horizons project were provided that could involve bias toward patients or prescribers. Four pharmacies stated that they were not taking new customers. No additional explanation was provided (e.g., “We're not taking new customers.”). Two pharmacies stated they were not accepting prescriptions from telehealth appointments. Finally, one pharmacy stated they required all the patient's medications be filled by them.

### 5.3 Payment and logistics

Three pharmacies stated that they did not accept credit card information provided over the phone as payment for medication. One of the pharmacies mentioned that they had experienced previous issues with accepting credit card payments over the phone due to the credit card being canceled. Another explanation was that the credit card could be stolen.

Three pharmacies stated that they did not carry the specified medication. One of these pharmacies stated that they had “a lot of problems” and that the “police had got involved.” Additional details about this were not provided. Finally, one pharmacy stated that they were out of stock of the specified medication.

## 6 Pharmacies that agreed to collaborate with the horizons project after follow-up

Overall, all reasons for the revised decision involved receiving more information about the Horizons project. The most common reasons pharmacies agreed to collaborate with the Horizon project were either (1) a therapeutic family court representative who referred an enrollee to Horizons contacted the pharmacy and explained the situation to the pharmacist or (2) brochures were mailed to the pharmacy about the Horizons project. Each of the two reasons occurred with three pharmacies. Additionally, two pharmacies agreed after a Horizons project member spoke with a pharmacist. One pharmacy agreed after a Horizons project member spoke with the pharmacy owner. For one pharmacy, the pharmacist reported knowing the patient whose medications would be paid for and agreed to accept credit card payment over the phone. No clear pattern emerged in the initial reasons for declining collaboration among pharmacies that later agreed. The most frequently cited initial barriers were from all categories and included not accepting new patients, concerns about exceeding dispensing limits, and not accepting credit card payments over the phone.

## 7 Discussion

MOUD is the front-line treatment for OUD, an illness that can result in a significant number of overdose deaths and major economic costs. Although initiatives, such as the Congressionally funded Mississippi Horizons Project, can help improve access to MOUD, barriers can limit patient access to these medications that are dispensed by community pharmacies. In the current project, almost two-thirds (65.1 %) of the 43 contacted pharmacies initially declined collaboration with the Horizons project. Ultimately, over half (53.5 %) agreed to collaborate with the project by accepting payment and dispensing the MOUD to the patient. Overall, pharmacists' reluctance to collaborate with the Horizons project was driven by regulatory misperceptions, unfamiliarity with patients and prescribers, and payment and logistical barriers. These reasons for declining collaboration, as well as the reasons a subset of pharmacies agreed to collaborate after additional follow-up, have important implications for stakeholders who collaborate with pharmacies to increase access to MOUD.

### 7.1 Regulatory misperceptions

The most common barrier to collaboration cited by pharmacies was having reached the allowable dispensing limit for buprenorphine. This is consistent with existing research demonstrating pharmacist concern and confusion about DEA regulations and wholesaler-imposed limits on MOUD.^32^ In response to regulatory scrutiny, pharmacists may misperceive wholesaler limits on MOUD as mandates from the DEA. This can make them feel uncomfortable dispensing MOUD.^33^ There is, however, no DEA limit on how much buprenorphine can be ordered and stocked.^25^ A perceived limit on dispensing MOUD could interrupt or limit access to treatment, increasing the risk of adverse outcomes, including overdose death, especially among underserved groups that encounter pre-existing structural and social disadvantages, including women, Black individuals, and individuals in rural areas.^34^ Pharmacists can write a letter to the wholesaler requesting an increased limit; however, they may be unaware that this option exists, may not know how or not feel confident to request a limit increase, or may perceive this process as too time-consuming.^32^

Educational interventions could help address misperceptions about MOUD prescribing regulations. There is a need to develop training specifically for pharmacists that reviews regulations. Funded programs, such as the Horizons project, and federal, state, and local agencies could improve the provision of up-to-date information on prescribing policies. The provision of this information, such as region-specific information from the state board of pharmacy, could increase familiarity with MOUD and policies on dispensing. This information may even help to develop more MOUD-friendly policies, increasing access to MOUD for patients. Personalization of educational materials based on a needs assessment of the pharmacies should be considered.^35^ Additional factors such as pharmacist availability and willingness to receive an educational intervention could also be beneficial to assess.^33^ Ultimately, these efforts across stakeholders at various levels could increase pharmacist collaboration with programs such as Horizons.

Another reason for denying collaboration was that prescribers needed to be local, or the prescriber was too far away from the pharmacy. A national survey found that 37.4 % of pharmacists shared this concern, especially among independent pharmacies.^36^ There is, however, no federal requirement that MOUD prescriptions must be from local prescribers. Explanations from pharmacies in the sample for filling prescriptions only from local providers included being unfamiliar with the prescribing patterns of non-local providers and concerns about the DEA. Unfamiliarity with prescribing patterns highlights concerns about potential misuse of prescribing privileges.^36^ This could consequently raise concern for the pharmacy about receiving DEA or state board oversight or an investigation if suspicious patterns of prescribing are suspected. There are, however, methods in place, such as the prescription drug monitoring program, that can help pharmacies to dispense MOUD without receiving unwarranted scrutiny from outside regulatory agencies. Finally, strengthening communication channels and the frequency of communication between pharmacists and prescribers helps decrease delays and resolve other barriers for MOUD dispensing.^36^ A combination of existing drug monitoring programs and increasing communication between parties involved in prescribing and dispensing MOUD could increase the likelihood of MOUD being dispensed and ameliorating any delays.

### 7.2 Unfamiliarity with patients and providers

Four pharmacies stated they were not accepting new patients, and pharmacists did not provide any additional explanation for this reason. Pharmacists can choose to accept or deny prescriptions. Operational concerns, such as staff shortages, may serve as an explanation for not accepting new customers. Some pharmacies may not accept new patients seeking MOUD due to misperceptions about dispensing limits for these medications, as previously discussed. Stigma may also have played a role in denying collaboration with the Horizons project. Existing research about views on MOUD also suggests that pharmacists can hold negative views toward MOUD that can even result in discrimination toward patients prescribed MOUD.^26^^,^^27^^,^^32^ Pharmacists can hold a persistent belief that MOUD is replacing one substance with another.^37^ Further, biases from providers that view people who use substances as untrustworthy and likely to misuse medications reinforce negative attitudes about MOUD.^37^ Some pharmacists may refuse to interact with patients who are prescribed MOUD.^38^ Personal moral or religious beliefs may play a role in dispensing MOUD, as research has documented these beliefs as influencing willingness to prescribe MOUD^39^ and other medications (e.g., emergency contraceptives).^40^ Consequently, pharmacists in the current study may have stated that they were not accepting new customers to avoid filling MOUD prescriptions. Simultaneously, these pharmacists may have accepted new customers with non-MOUD prescriptions, although the Horizons project did not assess this.

Similarly, pharmacies declined to fill prescriptions issued through telehealth appointments despite this not being a requirement for filling a prescription. There is no way to determine whether a prescription was obtained in-person or through a telehealth appointment.^41^ Additionally, one pharmacy stated they required all the patient's medications to be filled at their pharmacy. This is also not a requirement for dispensing MOUD. Pharmacies would be unable to verify if all medications prescribed to the patient were being filled at their location. While these reasons may be related to regulatory misperceptions regarding MOUD, perceptions of mistrust regarding MOUD (e.g., misuse or diversion of MOUD)^37^ likely played a role in providing these reasons in a refusal to collaborate with the Horizons project.

Efforts to address MOUD by increasing familiarity and reducing stigma, including educational interventions even among pre-licensed health care professionals, appear promising.^39^^,^^42^ Educational interventions may be especially effective for increasing the favorability of MOUD among pharmacists who are less familiar with MOUD.^43^ Stakeholders can help address stigma by providing information to pharmacists about MOUD and the prescribers of these medications. This could include information and the development of trainings for pharmacists about telehealth providers and protocols, and subsequent support for understanding telehealth prescribing procedures. Telehealth providers help provide patients with services that may otherwise be difficult to access, especially in rural areas. This understanding may help increase collaboration by helping pharmacists become less suspicious of non-local prescribers and be more willing to accept patients who are prescribed MOUD including via telehealth prescribers. Stakeholders, such as state pharmacy boards, could monitor and provide incentives to pharmacies that dispense MOUD. The development of these MOUD-friendly policies for pharmacies could help normalize and encourage stocking and dispensing of these medications.

### 7.3 Payment and logistical barriers

Finally, payment and logistical barriers contributed to denying collaboration with the Horizons project. Regarding payment, three pharmacies stated they did not accept credit card payment over the phone. Two of these pharmacies expressed concerns about the possibility of fraud (e.g., cancellation of the card). Although these concerns are justifiable, pharmacies still refused to collaborate despite the legitimacy of the Horizons project that could pay for and monitor patients who were prescribed MOUD. This refusal to accept payment can further limit financially disadvantaged patients' access to MOUD.^29^ Logistical reasons for declining collaboration included not carrying the specific MOUD and the medication being out of stock. These concerns could be addressed by ordering the MOUD from the wholesaler, which usually arrives within 24 h. Educational interventions similar to interventions to address regulatory misperceptions could increase willingness for pharmacies to collaborate with stakeholders. Further, regulatory bodies from federal to regional levels can help create an environment that allows for and encourages the stocking and dispensing of MOUD. This could include financial support and incentives,^44^ operational flexibility,^45^ and regulatory adjustments.

### 7.4 Pharmacy collaboration after initial denial

Eight (18.6 %) pharmacies did not initially collaborate with the Horizons project but joined after follow-up. Overall, pharmacies agreed to collaborate with the Horizons project due to a community member involved in the enrollee's treatment through therapeutic family court consulting a pharmacist, or due to brochures with information about the Horizons project being mailed to the pharmacy. Establishing and leveraging existing collaborative relationships with pharmacies and other stakeholders and providing additional information about the legitimacy of the Horizons project ultimately increased pharmacies' willingness to collaborate. Peer-to-peer engagement is an effective strategy to decrease stigma and increase pharmacist willingness to dispense MOUD.^32^ Follow-up contact from Horizons project team members with pharmacies likely influenced willingness to collaborate and dispense MOUD, especially since increased communication between parties involved in MOUD treatment improves MOUD dispensing behaviors.^36^ Future efforts to establish collaboration with independent pharmacies could benefit from involving prescribers, especially if prescribers are not local or prescribe via telehealth appointments. Communication between pharmacists and prescribers can help build trust about prescribing patterns.^46^

### 7.5 Limitations

Limitations to the current findings should be noted. First, the selection of pharmacies was limited to pharmacies near patients who were eligible to have financial costs covered by the Horizons project. Due to pharmacies not being randomly selected, the results of the current project may not fully represent MOUD dispensing practices throughout Mississippi. The experience may also not generalize to pharmacies in urban areas or other states, which may vary in MOUD access and dispensing practices.^34^^,^^47^ The project about this experience was based on contact notes from phone calls with pharmacies. There was no formal interview structure during phone calls, and transcripts or audio recordings of phone calls were not kept. Double coding, formal or secondary thematic coding, or validation did not occur. Thus, a formal coding process and a second coder were not used. Single-person categorization was used. Thus, there is some degree of subjectivity in interpreting the pharmacy contact notes, although a degree of subjectivity is a common limitation of qualitative research.^48^ Future studies and pharmacy collaboration efforts can use the findings from the current study to develop more structured interview questions to improve documentation of explanations for collaborations. Enhanced qualitative research methodologies such as this could help verify the findings for the current study and increase the reliability of findings across contexts.

## 8 Conclusion

Collaboration with independent pharmacies allowed the Horizons project to provide and pay for MOUD for patients with financial limitations. However, nearly half of contacted pharmacies ultimately did not participate, and pharmacist non-collaboration can directly disrupt treatment access. Through initial contact and follow-up with pharmacies that did not initially or ultimately agree to collaborate, several key lessons were learned that are applicable for future pharmacy collaboration efforts, especially in rural areas and Southeastern states. MOUD collaboration efforts involved a complex interplay of regulatory (e.g., misperceived limits on MOUD dispensing), logistical (e.g., not accepting credit card payment over the phone), and familiarity and likely stigma-related (e.g., not taking new customers without explanation) factors.

While educational interventions can help address knowledge gaps, they may be insufficient to ensure sustained collaboration. Efforts to foster lasting improvement could involve aligning education with supportive policy, system-level changes, and stronger communication between prescribers and pharmacies. This can begin by identifying and addressing legal and procedural misunderstandings about MOUD dispensing and wholesaler limits, followed by region-specific, board-endorsed training that clarifies regulatory directives. Building on this foundation, small-scale partnerships with pharmacies willing to fill MOUD prescriptions from telehealth providers can be tested to model how integrated prescriber–pharmacist communication facilitates safe and efficient dispensing. Finally, sustained collaboration will depend on creating an environment where stocking and dispensing MOUD is both feasible and encouraged, which may require targeted financial support, operational flexibility, and regulatory adjustments.

Integrating education with structural and policy support, while nurturing collaborative partnerships with pharmacies and other key stakeholders, can help increase familiarity, reduce stigma, address operational challenges, and enhance cooperation. This comprehensive, multifaceted strategy holds the strongest promise for bridging the treatment gap and improving MOUD access, ultimately leading to better patient outcomes and saving lives nationwide.

Funding: The project described was supported by Grant Number H79FG000787 from the Substance Abuse Mental Health Services Administration. Its contents are solely the responsibility of the authors and do not necessarily represent the official views of the Substance Abuse Mental Health Services Administration.

## CRediT authorship contribution statement

**John M. Vasko:** Writing – review & editing, Writing – original draft. **Erin Shirley Orey:** Writing – review & editing. **Carolanne Wartman:** Writing – review & editing. **James K. Rowlett:** Writing – review & editing. **Jefferson D. Parker:** Writing – review & editing. **Donna M. Platt:** Writing – review & editing. **Julie A. Schumacher:** Writing – review & editing.

## Declaration of competing interest

The authors declare that they have no known competing financial interests or personal relationships that could have appeared to influence the work reported in this paper. The project that allowed us to write this manuscript was supported by Award number: H79FG000787 from the Substance Abuse Mental Health Service Administration.
